# Anthropology from the Margins: The Craniological Network of Carl Gustav Carus

**DOI:** 10.1007/s00048-025-00423-7

**Published:** 2025-08-14

**Authors:** Stephan Strunz

**Affiliations:** Institut für Geschichte der Medizin, Technische Universität Dresden, Dresden, Deutschland

**Keywords:** Physical anthropology, Human remains, Anatomy, Craniology, Anthropologie, Human remains, Anatomie, Kraniologie

## Abstract

This paper examines the role of Carl Gustav Carus within the emerging field of physical anthropology during the mid-nineteenth century. Despite his extensive connections with contemporary anthropologists, Carus’s craniological approach was increasingly marginalized, ultimately excluding him from the foundational phase of German anthropology. Reconstructing Carus’s craniological network from largely unpublished correspondence, this study presents four key aspects. First, it traces the scholar’s research in craniology, situating it within Goethe’s and Oken’s morphological vertebrae theory. Second, it outlines the typology and transnational scope of his craniological network. Third, the paper explores Carus’s complex relationship with anthropology, highlighting the tension between his physiognomic methods and the evolving anatomic standards of his time. Lastly, the analysis highlights his aristocratic approach to anthropology, characterized by selective access to his cranial collection and an emphasis on the European “genius” as the pinnacle of human development. Ultimately, although well-connected among his peers, Carus was marginalized as physical anthropology shifted toward more empirical, metrical-morphological methodologies.

**Supplementary Information:**

The online version of this article (10.1007/s00048-025-00423-7) contains supplementary material, which is available to authorized users.

## Abstract (de)

In diesem Beitrag wird die Rolle von Carl Gustav Carus für die Formationsphase der Anthropologie in der Mitte des neunzehnten Jahrhunderts untersucht. Trotz seiner weitreichenden Verbindungen zu zeitgenössischen Anthropologen wurde Carus’ kraniologischer Ansatz an den Rand gedrängt, was ihn letztlich aus der Gründungsphase der deutschen Anthropologie verbannte. Die Studie rekonstruiert Carus‘ kraniologisches Netzwerk durch größtenteils unveröffentlichte Briefe und stellt vier zentrale Aspekte vor. Erstens zeichnet sie Carus’ wissenschaftliche Entwicklung in der Kraniologie nach und ordnet sie in Goethes und Okens morphologische Wirbeltheorie ein. Zweitens wird die Typologie und die transnationale Reichweite von Carus’ kraniologischem Netzwerk skizziert. Drittens wird Carus’ komplexe Beziehung zur Anthropologie untersucht und das Spannungsverhältnis zwischen seinen physiognomischen Methoden und den sich entwickelnden anatomischen Standards seiner Zeit herausgestellt. Schließlich unterstreicht die Analyse Carus’ aristokratischen Ansatz in der Anthropologie, der durch den selektiven Zugang zu seiner Schädelsammlung und die Betonung des europäischen ‚Genies‘ als Höhepunkt der menschlichen Entwicklung gekennzeichnet ist. Mit der zunehmenden Fokussierung auf metrisch-morphologische Methoden wurde Carus, obwohl exzellent vernetzt, zunehmend aus anthropologischen Diskursen herausgeschrieben.

## A Deliberate Exclusion

In September 1861, a group of anatomists, physiologists, and zoologists gathered in Göttingen to discuss methodological standards for the emerging field of physical anthropology.^1^ The event was hosted by Karl Ernst von Baer (1792–1876) and Rudolf Wagner (1805–1864), purposely excluding a prominent figure of German naturalism, Carl Gustav Carus (1789–1869). Despite his extensive contributions to craniology, physiognomy, and racial science over more than two decades—making him a seemingly suitable participant for a convention focused on methods for measuring human crania (Ottow 1966: 45–46)—Baer had made it clear beforehand that Carus would not be welcome. Baer’s antipathy toward Carus is most evident in his correspondence with the Swedish anatomist Anders Retzius (1796–1860), who had introduced the influential cephalic index in 1841 (Fish et al. 2024: e35–e36) and was a key figure in international craniological debates (Blanckaert 1989). In a letter to Retzius in 1858, Baer wrote, “Third, I must inform you that Carus, whom I had not contacted at all, has written to me. I shall have to answer him. Now it will hardly be possible, although I am not yet doing so, to conceal the meeting from him for the time being. You do not seem to wish him to come, nor do I for that matter … he rides too much on old carriage horses.”^2^ Despite acknowledging that excluding Carus, given his reputation, might risk “turning him into an enemy,”^3^ Baer and Wagner ultimately decided to proceed with their decision as preparations for the meeting advanced in early 1861.^4^

In this way, Carus was sidelined from what would become a foundational moment of German anthropology, a convention that paved the way for the subject’s institutionalization in German-speaking academia (Hossfeld 2005: 87). This exclusion merits closer examination. At the age of 72, Carus was indeed old, but many of the participants, including Baer himself, as well as the anatomists and physiologists Ernst Heinrich Weber (1795–1878), Carl Georg Bergmann (1800–1874), and Willem Vrolik (1801–1863), were of the same generation. Moreover, Carus was both universally recognized and highly connected among naturalists, as evidenced by his election as president of the *Deutsche Akademie der Naturforscher Leopoldina* in 1862 (Lienert 2009). However, unlike the Göttingen group, Carus remained committed to a methodological approach considered archaic by the 1860s. His anthropological writings on craniology were overshadowed by a Romantic framework, which sought to interpret empirical findings through analogies and symbolism. For contemporaries such as Baer and Wagner, Carus’s *naturphilosophisch *foundation had become deeply unappealing. Wagner, expressing his admiration for Baer shortly before the conference, remarked, “Among our anthropological peers, who range between the narrow-minded à la Gruber and the fantasists à la Carus, only your approach satisfies me.”^5^

This paper, then, aims to examine the paradoxical position of an intellectual outsider who nevertheless was very well connected to leading scholars in the field. The history of racial science is frequently portrayed as the inevitable progression of a unified, unchanging doctrine. This paper, however, reveals that craniology—like any other scientific field—was marked by internal divisions and significant epistemic breaks. While scholars of Carus and Romantic medicine have noted his contributions to mid-nineteenth century craniology and racial science (Gray 1999; Hagner 2009; Melzer 2009a; Richards 2020), Carus is largely absent from historiographies of anthropology (Glenn Penny 2008; Hossfeld 2005; Hannaford 1996; Querner 1986). Michael Banton has noted that Carus treated the topic of race in nine books, yet argues that it played a relatively minor role in his overall oeuvre (Banton 2002). Almost a century ago, Eric Voegelin praised Carus as the first to offer anthropology an organic theory of race that linked mind and body (Voegelin 1933; Wund 2019). For an earlier generation of historians of race, Carus appears to have been a significant figure, even a model of an alternative conception of racial science. While this article does not endorse Voegelin’s positive evaluation in any sense, his assessment nonetheless highlights that Carus was once regarded an important—if now largely forgotten—contributor to racial science. Recent scholarship reinforces this view, demonstrating that racial themes occupied a much broader portion of Carus’s work and that he actively sought to position himself within international debates on race during the 1840s and ’50s (Strunz et al. 2025).

This paper seeks to contribute to this scholarship by providing a more detailed analysis of Carus’s craniological network. As I will show, the network that Carus relied upon to acquire his craniological collection of 300 skulls, plaster casts, and face masks included many champions of early anthropology such as Daniel Friedrich Eschricht (1798–1863), Joseph Barnard Davis (1801–1880), Anders Retzius, John Thurnam (1810–1873), and even Baer and Wagner. In addition to those with private collections, Carus’s network also comprised various naturalists and physicians who acted as intermediaries between him and different institutions or private individuals. As such, his craniological network did not differ much from the networks of (in)famous skull collectors such as Samuel George Morton (1799–1854) (Fabian 2010: 36) and Retzius (Ljungström 2014: 169). Carus not only used these connections to acquire new materials but also promoted his published works on race and craniology, albeit with limited success.

While prominent scholars such as Baer and Wagner sought to marginalize him within the emerging discipline, others acknowledged certain aspects of his methods. One might assume that the criticism directed at Carus was, at least in part, due to his interpretation of human crania. Whereas mid-nineteenth German anthropology was largely monogenist in design (Glenn Penny 2008: 79), Carus used his data to support a white supremacist, polygenist ideology. The disagreement, however, stemmed less from his political stance, and more from the physiognomic framework within which it was embedded—a framework that had become increasingly unfashionable. Carus’s outsider status is further highlighted by the unscholarly figures within his network. His correspondence with members of courts and the upper bourgeoisie reveals that he understood craniology as an aristocratic pursuit, whose results were reserved for the privileged few. Carus was appalled by public displays of human crania and maintained strict confidentiality around his private collection, offering access only to members of the upper classes during private tours that he personally conducted.

Drawing on his published works, memoirs, and unpublished correspondence from various university collections, this paper explores four aspects. First, I provide a brief overview of Carus’s scholarly trajectory in the field of craniology, offering a relevant historiographical context. Second, I sketch out a typology of Carus’s craniological network by describing its composition and the main skull donors. Many of his suppliers were mutual members of the Leopoldina Academy, highlighting Carus’s skill as an intellectual networker. Third, the paper delves into the ambiguous relationship between Carus and the emerging field of anthropology. Although many contemporaries openly opposed his approach, they nevertheless engaged in extensive discussions with him about exchanging crania. It seems that the ever-pressing question of skull acquisition often outweighed intellectual concerns, leading scholars with opposing views to pragmatic collaborations. Fourth, Carus’s somewhat outsider position becomes more evident when considering his exchanges with non-anthropologists to whom he showcased his cranial collection. These interactions reflect an aristocratic attitude toward science, which, in Carus’s view, was a pursuit reserved for noble minds alone.

## Carus’s Craniological Trajectory

Like many of his contemporaries who would venture to become representatives of early anthropology, Carus had a background in comparative anatomy. His intellectual development was deeply influenced by Romantic *Naturphilosophie*, particularly the “osteological program” (Rupke 2009 [1994]: 124) of Johann Wolfgang von Goethe (1749–1832) and Lorenz Oken (1779–1851). Goethe popularized the concept of the archetype (*Urtyp*) to account for morphological features found in all living organisms (Rehbock 1990: 146). In the 1790s, Goethe’s search for morphological archetypes led him to examine animal vertebrae, identifying “an inclusive form … that would contain all of the parts really exhibited by the range of different vertebrae species” (Richards 2002: 443). Both Goethe and Oken proposed that the bones of the skull could be viewed as more advanced forms of vertebrae, providing a new avenue for the analysis of archetypical structures (Richards 2002: 492). Building on Goethe’s idea of six archetypical skull vertebrae, Carus conducted his own research on comparative anatomy. In his 1828 work *Von den Ur-Theilen des Knochen- und Schalengerüsts *(On the Archetypical Pieces of Bone and Shell Structures), which he sent to Goethe for review (Carus 2001), Carus not only posited that the human brain was the most developed among all species but also directly linked brain development to the formation of skull vertebrae. He proposed that three masses of the brain (cerebellum, corpus quadrigemina, hemispheres) corresponded to three skull vertebrae (roughly equivalent to the occipital bone, parietal bones, frontal bone), which, in turn, were associated with three mental faculties (will, feeling, cognition). This finding was in line with Oken’s vertebrae theory. Consequently, Carus considered the skull as a sign or symbol of the degree of perfectibility of the brain (Carus 1828: 176–77).

At this stage, Carus had not yet made connections between cranial development and race, although he clearly regarded the human cranium as the pinnacle of organic development. It was not until 1835 that race entered the picture (Banton 2002: 35; Bräutigam 1990: 133). A possible catalyst for this shift could have been Carus’s extended journey to Paris and the Rhineland, where he accompanied the Saxonian king as a personal physician. During his stay in Paris, Carus visited Cuvier’s Galerie d’Anatomie comparée du Muséum national, gaining access to a private exhibition chamber. This room housed a variety of specimens, including the remains of Sarah Baartman, a cast of Franz Joseph Gall’s skull, and the “squashed, curious skulls of ancient Peruvian mountain dwellers” (Carus 1835: 180). Peruvian skulls would remain a life-long obsession of Carus (Carus 1842, 1856, 1863a), possibly sparking the racialization of his craniological thinking. On his return to Germany, Carus stopped in Göttingen to see Johann Friedrich Blumenbach (1752–1840) and his craniological collection, which was considered one of the largest and most comprehensive collections of the 1830s (Marino 2021: 74). Once again, Carus was struck by the sight of “squashed” Peruvian skulls, but this time he found them not merely “curious” but also “disgusting” (Carus 1836: 276). Carus also expressed interest in other non-European skulls such as those of ancient Egyptians, Malays, and Kalinagos.

Beginning in 1838, Carus outlined a racialized theory of anthropology in his three-volume work *System der Physiologie *(System of Physiology) (Carus 1838). The third volume, published in 1840, firmly tied craniology to racial science. As numerous scholars have analyzed the features of his race theory (Gray 2004; Husmann 2010; Müller-Tamm 1995; Rupke 2018; Stubbe 1989), I will only briefly outline its main components here. Unlike contemporary anthropologists such as Blumenbach, Tiedemann, Baer, or Wagner, Carus embraced a polygenist racist theory of human difference (Rupke 2018: 239). He claimed that each race had developed independently in distinct climatic zones, leading to variations in its mental capabilities. Carus aligned the climatic differentiation of human races with analogies on the basis of the diurnal cycle of the sun. He categorized humans into four races: “people of the eastern twilight” (Chinese, Mongols, Malaysians), “people of the daylight” (Europeans, Indians, North Africans), “people of the western twilight” (Indigenous peoples of the Americas), and “people of the night” (sub-Saharan Africans, African Americans). The quadripartite division of race also corresponded to an analogy with the four organic realms (proto-organic, plant, animal, and human) and their progressive perfection (Carus 1838: 119–22). European people, as “people of the daylight,” epitomized the highest degree of human development, illustrating his belief that organic beings tended toward formal perfection, which was evidenced by the famous “face angle of 90 degrees” allegedly found in ancient Greek skulls—an idea he had adopted from Peter Camper (Carus 1838: 126–27; Querner 1986: 290).

Curiously, the first volume of *System der Physiologie* did not use craniology to establish racial hierarchies. Carus began to rely more heavily on this method only in the third volume. The combination of “soft” anthropological speculation with “hard” anthropometrical data was likely driven by the establishment of Carus’s skull collection. In this third volume, Carus compared the cranial dimensions of Schiller and Napoleon, two presumed “geniuses” with large frontal bones, to those of an enslaved African, a Fijian, and a Surinamer, arguing that these measurements allowed for “curious analogies with the mental abilities of these persons.” Unsurprisingly, the smaller skulls of non-Europeans were presented as evidence of supposedly inferior intellectual capacities, thus reinforcing the racial hierarchies suggested in the first volume of *System der Physiologie *(Carus 1840: 344–45).

In the 1840s, Carus continued along this craniological trajectory, publishing several works on the basis of his expanding collection of cranial specimens. Following his 1841 textbook on a “new cranioscopy”—a term highlighting the morphological aspect of his skull science—Carus produced two illustrated atlases that provided readers with profile drawings, metrical data, and explanatory notes on selected objects (Sommer 2024; Carus 1843–1845, 1864a). Although the term “cranioscopy” was originally coined as a synonym for Franz Joseph Gall’s (1758–1828) phrenological project (Anon 1805), Carus redefined it in his “new cranioscopy,” intending to counter Gall’s “untenable hypotheses … reveries and delusions” (Carus 1841: 9). Consistent with his vertebrae theory, Carus argued that the form of the skull indicated only the “fundamental directions of the soul” (Carus 1841: 9) rather than the specific moral qualities suggested by Gall.

As Michael Hagner has observed, the arrangement of crania within these atlases reflected Carus’s idiosyncratic approach toward anthropology, blending racial science with hagiography. The skull drawings of Western “geniuses” and statesmen were intended for bourgeois and aristocratic audiences familiar with classical sculpture, and they visually contrasted with the allegedly inferior aesthetics of abnormal or deviant subjects, as well as non-European people, an approach inspired by the works of Johann Friedrich Blumenbach and Petrus Camper (Hagner 2009: 267; Martin 2021: 73). The 1849 commemorative publication marking Goethe 100th birthday, *Ueber ungleiche Befähigung der verschiedenen Menscheitstämme für höhere geistige Befähigung *(On the Unequal Aptitude of Various Human Races for Higher Intellectual Development), widely regarded as the quintessential expression of Carus’s racism (Gray 2004; Stubbe 1989: 45; Wund 2019: 9), only synthesized views Carus had been advocating since 1838. The dedication to Goethe once again highlighted the indebtedness of Carus’s race theory to Goethe’s and Oken’s morphological approach, and its increasing emphasis on craniology. This emphasis became even more pronounced in the 1850s, when Carus expanded from crania to other skeletal elements, considering all features of the human shape (*menschliche Gestalt*) as potential indicators of racial identity (Carus 1853, 1854). His craniological work thus culminated in a generalized physiognomy of race (Müller-Tamm 1995: 132).

## A Brief Description of Carus’s Craniological Network

For more than three decades, from 1835 to 1867, Carus obtained a collection of more than 300 crania and plaster casts of skulls, heads, and face masks (Melzer 2009c: 317). He categorized these objects according to his own racial classification system: 215 objects were attributed to “people of the daylight,” 45 to “people of the eastern twilight,” 23 to “people of the western twilight,” and 17 to “people of the night.” This distribution evinces that the bulk of his collection consisted of objects from Europe and North Africa. Unlike craniological objects of European origin, which were mainly comprised of casts, most craniological objects of non-European people were actual human remains (Melzer 2009b: 257). During Carus’s lifetime, he housed the collection in his private residence in Dresden. After his death in 1869, it was transferred to the zoological collection at the University of Leipzig, where it was relocated several times over the next century. Today, one part of the collection is housed by the Institute of Anatomy at Leipzig University, whereas the other part has been incorporated into the anthropological collection of the Dresden Museum of Ethnology (Melzer 2009b: 258). After exploratory work by Kathleen Melzer and Petra Martin (Martin 2021; Melzer 2009b, c), provenance research and potential restitution is currently facilitated through a project funded by the Deutsches Zentrum für Kulturgutverluste at the Institute of Anatomy at Leipzig University.^6^

For this paper, I analyzed Carus’s published works and correspondence, memoirs, and unpublished letters from hundreds of administrators, anatomists, anthropologists, naturalists, physicians, zoologists, and private individuals.^7^ Additionally, I consulted correspondence from the private archives of Karl Ernst v. Baer, Alexander v. Humboldt (Ette and Knobloch 2009; Schwarz 2009), and Rudolf Wagner.^8^ These sources offer detailed insights into the collection practices of a highly connected scholar who nonetheless remained an outsider to early physical anthropology. The sources allowed me to trace the collection networks for approximately 40 objects in the Carus collection. To identify potential objects, I cross-referenced these sources with the two editions of the collection’s catalogue that Carus published during his lifetime (Carus 1863b, 1867 [1863]). An open-data Excel spreadsheet is provided alongside this paper, containing more specific information on the objects. In addition to catalogued objects, the spreadsheet includes skull offers and requests, opening a broader perspective on Carus’s craniological network. Importantly, the sources provide only fragmentary insights into his collection network. A significant gap and distortion arise from the fact that skulls and plaster casts obtained through commercial networks, such as the nine Asian skulls he reportedly brokered from a Leipzig trader in 1841 for 125 thalers (Melzer 2009b: 256), are largely absent from the correspondence. Nevertheless, since Carus’s correspondence spans a wide range of contacts, it can be assumed to be representative, allowing for inferences about the typology of his craniological network.

In her documentation of Carus’s craniological collection, Melzer has found that Carus did not acquire any skulls before 1837. According to her research, his first acquisition was a plaster cast of Friedrich Schiller’s presumed skull in February 1837 (Melzer 2009b: 317). However, correspondence with Gottlob Regis (1791–1854) suggests that Carus may have obtained casts even earlier, shortly after his trip to Paris in 1835. This indicates that his visit to Cuvier’s Galerie d’anatomie comparée likely served as the catalyst for his craniological endeavors. In a letter to Regis, Carus mentions obtaining a plaster head of Georges Cuvier himself, which might have sparked his fascination: “I received a splendid, large, even colossal head of George Cuvier from Paris, along with several others, and you should also take a look at this curious fellow” (Carus 1956 [1835]: 381r). Based on the 42 objects for which acquisition data are available, Carus gradually amassed a significant collection of craniological objects, with most acquisitions occurring between 1840 and 1860.

**Fig. 1:**
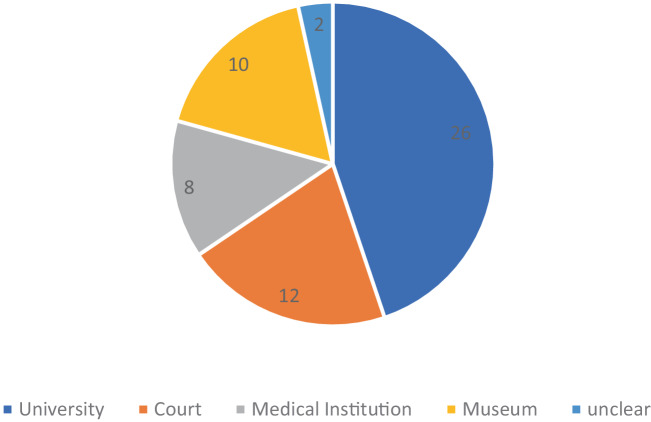
Objects by Interlocutor’s Institution

**Fig. 2:**
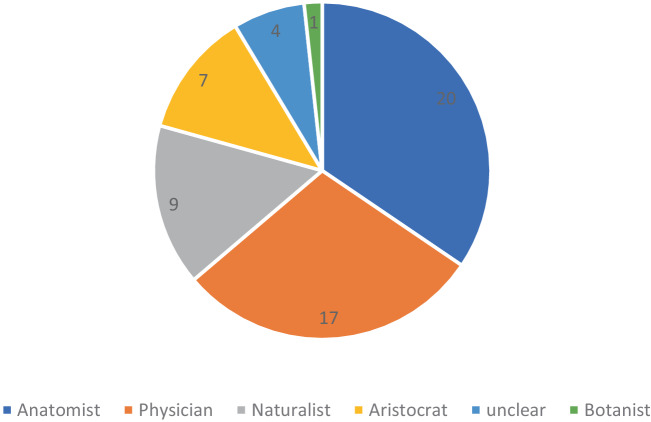
Objects by Interlocutor’s Profession

The sources reveal craniological materials obtained through 38 contacts. For most objects (42), the transactions materialized; for others (16) there were, at times, lengthy exchanges without tangible results. The majority of objects were held by interlocutors at universities (26), underscoring the scholarly nature of his craniological network (see Fig. 1). Classed by profession, they came from fellow anatomists, pathologists or physiologists (20) at universities, highlighting Carus’s strong integration into the “academic anatomical elite in Europe”—a trait he shared with the anthropologist and skull collector Anders Retzius (Ljungström 2014: 171). Other objects (9) came or were requested from contacts associated with medical facilities (hospitals, private practices, spas) or royal courts (12, see Fig. 2). Although commercial sources are missing from this assessment, the dominance of research institutions makes Carus’s network quite distinct from eminent skull collectors such as Samuel Morton, who relied mainly on doctors and military men affiliated with the Academy of Natural Sciences in Philadelphia (Mitchell and Michael 2019: 87). Unlike Morton, who obtained his skulls from “battlefields and graveyards in the Americas” (Mitchell and Michael 2019: 87), Carus’s objects were often already in the possession of other collectors. When Carus tried to obtain craniological material from scientific expeditions, the success rate was rather low. For example, when he attempted to obtain 16 skulls from the Novara expedition in 1863, the University of Göttingen’s zoological institute was able to offer the owner a better price.^9^

This might explain why most of Carus’s collection (162 objects) comprised plaster casts of skulls housed elsewhere, underscoring his deep connection to fellow academics and museums. Most members of his craniological network were based in Europe, predominantly in Germany, with other contacts in Austria, Denmark, England, Greece, Italy, Russia, Scotland, Sweden, and Ukraine. Among those who would make a name for themselves in the emerging field of anthropology were Karl von Baer, Joseph Barnard Davis, Johann Christian Gustav Lucae, Anders Retizus, John Thurnam, and Rudolph Wagner. The scholarly network behind Carus’s craniological collection highlights his skills as an intellectual networker, a trait that was partly due to his integration into various intellectual societies. Notably, 19 of his 38 contacts were fellow members of the Leopoldina Academy, which Carus would eventually lead as president from 1862 onwards.

These sources provide insights into his position within the emerging field of anthropology. Although it might seem odd to describe someone who was a recipient of various academic prizes and honors as an academic outsider, Carus was a marginal figure in the debates among early anthropologists. As I will show in the next section, this was less due to a lack of interest in his anthropological theories and more due to the prominence of the vertebrae theory, which paved the way for Carus’s increasing orientation towards physiognomic symbolism.

## An Ambiguous Position Within Early Anthropology

Opportunities for integration within anthropological discourses existed in the 1840s. When Carus embarked on his craniological work during this period, the field was far from being consolidated, encompassing anatomists, ethnologists, phrenologists, physiologists, and zoologists, with vibrant transnational exchanges (Poskett 2015: 266). Early anthropologists such as Retzius and Wagner engaged with Carus’s works in the 1840s but found it impossible to reconcile his theories with their own. By the 1850s, as disciplinary boundaries began to solidify and national traditions emerged (Kuklick 2008: 33–110), Carus was largely overlooked in anthropological discussions. For instance, although he publicly praised the so-called American School of Physical Anthropology with Samuel Morton and his followers (Carus 1840: 351, 1842: 1107, 1856, 1858), he was cited only once in the two volumes of *Types of Mankind *(Nott and Gliddon 1854) and *Indigenous Races of the Earth *(Nott and Gliddon 1857).

Based on Dietrich von Engelhardt’s comprehensive bibliography, Carus’s works on comparative anatomy and gynecology were extensively reviewed, often receiving 10 or more reviews, whereas his anthropological works received only limited attention, sometimes none at all. While *System der Physiologie, *possibly owing to its medical title, still attracted the interest of seven reviewers, the Goethe anniversary publication was only reviewed once (von Engelhardt 2023: 55–71). In 1863, the anthropologist Carl Vogt remarked that, concerning Carus’s vertebrae theory of the skull and its corresponding anthropological significance, “no one has yet followed him down this path” (Vogt 1863: 52). This assessment was not entirely accurate, as the anatomist Hermann Welcker pointed out a year earlier. In fact, the anatomists Johann Christian Gustav Lucae (1814–1885) and Emil Huschke (1797–1858) used Carus’s skull vertebrae as the fundamental units for measuring skulls until the 1860s (Welcker 1862: VIII). Nevertheless, Carus was aware of the overall rejection of his craniological writings, which he attributed to public confusion, claiming that people either perceived too much or too little of Gall’s phrenology in his work rather than assessing it on its own merits (Carus 1853: XIII–XX).

Browsing through some of the reviews offers a clearer picture of Carus’s unfashionable approach toward anthropological questions. After the publication of *System der Physiologie*, one reviewer criticized the work for focusing less on physiology and more on a misguided form of anthropology, a transgression “that happened with very little luck and would have been better left undone” (Leupoldt 1840: Sp. 69). The reviewer especially mocked Carus’s description of racial development following the “ideal pattern” of a “spiral line” (Leupoldt 1840: Sp. 68–69). Carus had indeed proposed that traits of less developed organisms were repeated in more advanced ones, much like a spiral (Carus 1838: 42–43). Regarding race, elements of the “people of the night” could be found among the lesser advanced “people of the daylight,” such as Indians, North Africans, and Turks (Carus 1849: 82). In reviewing Carus’s early works on cranioscopy, the physiologist August Franz Josef Karl (1787–1865) criticized Carus in 1843 for approaching the brain “from the standpoint of abstraction,” rather than through “single data of observation.” Karl argued that to measure the skull with a ruler was “to regard the soul organ as a wheel of cheese,” ignoring qualitative differences within brain matter. He also challenged Carus’s correlation between skull size and mental capacities, stating, “I have made many observations of men with significant mental powers whose skulls were far below average, in fact they were small” (Mayer 1843: 404). In the international press, his works fared no better. Phrenologists slammed Carus’s cranioscopic treatises of the early 1840s, which had been partially translated and published in the *Medical Gazette *(Carus 1844), for containing “flimsy analogies” (Anon 1842: 159). In 1862, following popularizations of his physiognomic works by English and French commentators, the *British Medical Journal *derided Carus’s “physiognomy of the human form” and cautioned against a growing “passion for the supernatural” (Brewster 1862: 566).

Focusing solely on reviews might give the impression that Carus’s anthropological works were dismissed outright. However, correspondence between Carus and other craniologists reveals a more complex picture. When Carus began assembling his collection in the early 1840s, fellow anthropologists still expressed curiosity in his craniological work. One of the first significant exchanges evolved in 1840 between Carus and the Danish anatomist Daniel Frederik Eschricht (1798–1863), a close acquaintance of Retzius. Eschricht, who had known Carus since 1826,^10^ was informed by the Dresden naturalist Ludwig Thienemann that Carus wished to exchange “the complete [works of Giuseppe Saverio] Poli for a number of specific natural historical objects.”^11^ The objects were Scandinavian crania and casts, which Carus wanted for his collection. In response, Eschricht dispatched a Greenlandic skull along with four casts of Scandinavian skulls. Praising Carus for his new intellectual endeavor, Eschricht expressed his willingness to assist “the celebrated Carus in any way in his work.”^12^ Six months later, Carus sent Eschricht a copy of his *Grundzüge einer neuen* … *Cranioskopie* and renewed his request for Scandinavian skulls. Eschricht directed Carus to the extensive collection of Retzius in Stockholm, whom he deemed more than willing to provide additional crania.^13^

Carus likely heeded Eschricht’s advice, as a letter from 1842 reveals an established correspondence between the two, with Retzius requesting the cast of an Avar skull from Carus.^14^ Three years later, Retzius informed Carus of a publication on the cerebral hemispheres in which he “mentioned [Carus’s] excellent views on the basic types of the brain” and expressed hope that Carus would “find [his] own views correct.” In this article, Retzius attempted to integrate his findings on the development of the cerebral lobes with Carus’s vertebrae theory of the skull. Retzius noted that in *Grundzüge einer neuen* … *Cranioskopie* Carus correlated the shape of the three skull vertebrae with the size and function of the hemispheres (cognition), the corpora quadrigemina (feeling), and the cerebellum (will) (Retzius 1845: 432–33). However, Retzius seemed to disagree with Carus, arguing that cognitive functions in humans were primarily located in the hemispheres and that “in humans the form of the skull is determined by the development of the three lobes of the hemispheres” (Retzius 1845: 433). According to Retzius, this contradiction could be resolved if one assumed that the location of all mental faculties in the hemispheres was merely a “higher repetition of the organs of will, feeling, and cognition”^15^ attributed by Carus to the three cerebral masses. A closer reading of Retzius’s work reveals that this reconciliation was possibly a polite attempt to conceal deeper inconsistencies. He later cited anatomical evidence that the cerebellum may not be the site of will and sex drive (Retzius 1848 [1847]: 240–41), as Carus had postulated.

That same year, the translation of Retzius’s theory of the cephalic index was published in Germany, introducing a novel method for categorizing race on the basis of “brachycephalic” (short-headed) and “dolichocephalic” (long-headed) types (Retzius 1845 [1842]). This new classification system offered a dynamic and flexible approach, focusing on “genealogical affinity” (Blanckaert 1989: 172) rather than geography or language. However, it also uprooted Carus’s equation of skull vertebrae, brain masses, and mental abilities. Since Retzius considered the occipital lobes of the hemispheres as the primary location of intellect, it was not large frontal but long occipital bones—found in Celtic and Germanic people—that corresponded to the highest intellectual abilities (Retzius 1848 [1847]: 244). In addition, Retzius’s racial classification contrasted with Carus’s system because it permitted the inclusion of both Europeans and Africans within the same (dolichocephalic) category (Blanckaert 1989: 175; Retzius 1845 [1842]: 88). In Carus’s framework, such a commonality would have been inconceivable, as he believed that Black people had smaller heads with short frontal and large occipital bones, traits he associated with reduced intellect and a tendency toward instinct-driven behavior (Carus 1841: 9–13).

The contrast between Carus and the emerging school of German anthropology is clearly illustrated in the way the two organizers of the Göttingen convention, Baer and Wagner, employed Retzius’s cranial classification scheme to promote their ideas of racial development. Unlike Carus, who emphasized static environmental influences, Baer and Wagner used the division between dolicocephalic and brachycephalic skull types to trace the distribution of races not through static environmental conditions, but through dynamic interaction and migration (Wagner 1861; von Baer 1860). Baer, for example, collected crania of ancient Rhaetian skulls from the Swiss canton of Grisons and sought to demonstrate that they displayed brachycephalic traits that sharply contrasted with the dolichocephalic skulls of nearby Alemannic Swiss populations. He argued that the Rhaetians, along with their Romansh-speaking descendants, originated from an early European brachycephalic group, largely supplanted by migrating dolicocephalic Celtic and Germanic people (von Baer 1860). In Churwalden, Baer found anatomical evidence of a blend of brachycephalic and dolicocephalic forms, which he interpreted as the “physical Germanization” of the Rhaetians (von Baer 1860, 247). His perspective suggested that race had to be considered not a higher principle but a hybridizing force mediated by history, language, and the body.

Despite these intellectual inconsistencies, Retzius’s attempt to reconcile Carus’s vertebrae theory with his own anatomic theory demonstrates how anthropologists at that time went to considerable lengths to integrate Carus’s craniological ideas into their own work. The spirit of rapprochement is further illustrated in Retzius’s offer to send casts from his “valuable collection of national skulls” to Dresden.^16^ In contrast, Carus did not reciprocate this intellectual generosity. As of 1853, Carus acknowledged that the “hemispheres are entirely covered by the frontal bone, partially by the parietal bones, and mostly by the occipital bone,” yet he maintained that this anatomical fact did not alter “the original relation and signature” of the parietal and occipital bones to the corpora quadrigemina and the cerebellum (Carus 1853: 113). He continued to assert that the hemispheres were exclusively responsible for cognition (Carus 1853: 117–18). Over time, Carus increasingly resorted to symbolism and analogical thinking, suggesting that the division of mental faculties along the three cranial vertebrae and three cerebral masses was inevitable since it signified the “importance of the number three” (Carus 1853: 62). With increasing mystification, Carus became increasingly alienated from the anthropological community. Retzius’s diminishing understanding of Carus’s work is reflected in his 1859 correspondence with Baer, where he lamented: “I do respect Carus, even though I cannot follow him in many ways.”^17^

In general, confounding anatomy and physiognomy were major obstacles to Carus’s integration into anthropological debates. A few years before the Retzius exchange, Rudolf Wagner, a co-organizer of the Göttingen convention, critiqued Carus’s *Grundzüge einer neuen Cranioskopie. *Wagner, too, focused on Carus’s strict association of intellect, feeling, and will with the hemispheres, the corpora quadrigemina, and the cerebellum. He argued that if Carus was correct, removing a frog’s cerebellum should eliminate its ability to move arbitrarily, and “excising the hemispheres would render any revelation of intelligence impossible.” However, such outcomes were contrary to experience. Wagner contended that “not only the fundamental faculties of the soul but also, the derived abilities depend on the totality of cerebral mass and not on specific parts” (Wagner 1842: 586).

After 1841, Wagner engaged extensively with Carus about craniological material. By May 1843, Wagner had received a copy of *Atlas der Cranioskopie *from Carus and expressed a desire to invite him to Göttignen to let him “oversee what is here.”^18^ Given the critical treatment of *Grundzüge einer neuen* … *Cranioskopie*, it is plausible that he saw a meeting as a chance to challenge and potentially expand Carus’s views. In any case, Wagner praised the qualities of Blumenbach’s collection for Carus’s purposes and looked forward to discussing various aspects of the collection with him. He even suggested a travel itinerary that would maximize efficiency and yield:

“Would it not be possible for you to make the short journey here from Dresden during these long summer days? You can be here from Halle in 24 hours by express coach. In one or two days you would have sampled our collection of approximately 250 skulls and recorded everything that you might wish to use in Dresden. I also hope that you will find some things here that you missed during your earlier stay” (in 1835).^19^

Carus declined the invitation, citing “too many ongoing activities” in Dresden. He expressed interest in reexamining the Göttingen collection at a later date, particularly the “*many* skulls of *important* and *very different* individuals” (emphasis in the original). Carus mused on how fascinating it would be to “compare skulls such as those of Luther, Napoleon, Göthe, Mozart with the heads of criminals!” If Wagner had any “important individuals” in his collection, Carus would be eager to receive a cast from their skulls.^20^ At the time, Carus was immersed in producing the second volume of *Atlas der Cranioskopie*, which focused on a grand physiognomic panorama of hagiographic, deviant, and racialized individuals, presenting “genius, madness, crime, gender, and race in a single context” (Hagner 2009: 265). In view of this, engaging in discussions with a colleague, who like Carus was busy “acquiring a craniological collection” of his own, might have appeared to be an unnecessary distraction from more important physiognomic matters.^21^

Carus’s increasing turn toward esoteric physiognomy likely explains why he was eventually dismissed from the founding moments of German anthropology. However, this shift might also shed light on why aspects of his work more compatible with the anthropological community were largely overlooked. Among those who did appreciate certain elements of Carus’s craniology was the Frankfurt-based anatomist Johann Christian Gustav Lucae. Like Baer and Wagner, Lucae participated in the Göttingen convention, where Baer reported on Lucae’s method of skull depiction (von Baer and Wagner 1861: 7; Lucae 1861).

By the late 1850s, anthropologists had called for the publication of illustrated catalogues to give fellow researchers access to foreign collections (Meigs Aitken 1858). Lucae welcomed these developments but warned against the use of “plastic renderings,” particularly free-hand drawings and photographs of skulls, as they systematically distorted the true object dimensions. He maintained that only geometric drawings were “capable of reproducing the most faithful and accurate image of nature on a flat surface” (Lucae 1861: 6). Without uniform geometric representations, meaningful comparisons between crania were virtually impossible. As a result, Lucae discarded the lithographic plates found in most of the famous craniological catalogues—including Blumenbach’s *Decas collectionis suae craniorum, *Morton’s *Crania Americana, *Nott’s *Indigenous *Races, and Davis’s *Crania Britannica*—as “scientifically useless.” These catalogues merely contained perspective drawings and failed to represent the crania either “strictly in profile” or “strictly in portrait view” (Lucae 1861: 7). Surprisingly, in addition to his own work, Carus featured as a role model in Lucae’s treatise. The skull drawings in the two volumes of *Atlas der Cranioskopie *were praised as rare examples of plates that met the “geometric” standard (Lucae 1861: 7).

As Carus’s correspondence shows, Lucae had been in contact with him as early as 1846, commending him for his accurate representations of crania. After perusing the second volume of *Atlas der Cranioskopie*, he described Carus’s skull drawings as “exceptionally beautiful, with some in the later sections unsurpassable.”^22^ So impressed was Lucae that he offered the skull of his late father, Samuel Christian Lucae (1787–1821), along with several casts of Hessian criminals, for inclusion in future volumes of *Atlas der Cranioskopie*.^23^ Carus reciprocated Lucae’s sentiments, as he had long been an advocate for geometrically precise skull drawings and had criticized Morton’s plates for their perspective distortions (Carus 1842: 1107). In 1864, Carus expressed “great joy” that Lucae “followed [my] rules precisely,” resulting in “useful representations for cranioscopic measurements” (Carus 1864a: X). What Carus did not see was that Lucae’s method was far more complex than his own. While Carus relied on clay impressions for his drawings, Lucae employed a self-designed diopter, the so-called Lucaesian apparatus, which afforded orthographic skull projections and was regarded as one of the finest craniological instruments at the Göttingen convention (Tammiksaar and Kalling 2018: 284).

In terms of anatomy, Lucae was one of the few followers of Carus’s vertebrae theory. Until the 1860s, he used the three Carusian skull vertebrae as basic units for his own measurements (Lucae 1854). Lucae’s praise for Carus’s methods made him an exception among the Göttingen group of anthropologists. While his measuring apparatus received critical acclaim, peers regarded Lucae’s decision to “measure the skull based on its vertebrae” (Welcker 1862: 22) as an unfortunate effect of Carus’s influence. Most anthropologists believed that using vertebrae as the foundational units of the skull resulted in measurements that were too imprecise for the exigencies of the discipline (Vogt 1863: 52). Herrmann Welcker, an anthropologist invited to the Göttingen convention, remarked in 1862: “Under all circumstances, however, the scope of Carus’ skull measures … is far too limited for any kind of in-depth analysis” (Welcker 1862: 22).

To a certain extent, Carus found a receptive audience in the Anglosphere, too. James Aitken Meigs, the custodian of the Morton collection in Philadelphia, was among those who favorably viewed the connection Carus drew between cranial form and mental characteristics, using it to “guide our examination of the different cranial formations, in their relation to psychical conditions” (Meigs Aitken 1857: 217). In 1863, Carus was admitted as a corresponding member of the London Anthropological Society, a short-lived polygenist and white supremacist organization led by James Hunt (Rainger 1978).^24^ There, he was invited to present a paper entitled “Construction of the Upper Jaw of the Greenlander,” in which he claimed to have found “a decided separation between the upper jawbone and the intermaxillary bone, almost as in children or in quadrupeds.” For Carus, if this characteristic was observed in a larger sample, it might indicate that so-called Greenlanders could “be classed among the lower order of human beings” (Carus 1864b: CXIV). The society’s rapporteur, Charles Carter Blake (1840–1897), disagreed. After examining relevant skulls in the Royal College of Surgeons and the British Museum, Blake “found that similar instances were sometimes present in other races than the Esquimaux.” He suggested that Carus’s assumption was likely due to anatomic sloppiness, as Carus had failed to differentiate between fissures in the “outer or inner side of the maxillary bone.” Fissures in the inner side, Blake noted, were not an “uncommon defect of ossification” and could occasionally be found in both Europeans and non-Europeans (Blake 1864: CXV).

Despite his marginal position in anthropological circles, Carus maintained contact with much of the anthropological world throughout the 1860s. While Baer was discussing Carus’s exclusion from the Göttingen convention with Retzius and Wagner, both were exchanging long letters with Carus about a potential swap of cranial material. Always eager to promote his work, Carus sent Baer a copy of his 1858 review of current anthropological literature, accompanied by a skull request.^25^ In response, Baer offered a range of non-European skulls as plaster casts, suggesting that they “swap head for head.” Baer even mentioned a preparatory trip to Göttingen where he planned to “discuss uniform ways of skull measurement” with Rudolf Wagner and others. For his way back from Göttingen to St. Petersburg, Baer suggested visiting Dresden to “meet a veteran and leader of natural history.”^26^ Other anthropologists, such as Joseph Barnard Davis, Giustiniano Nicolucci, and John Thurnam, also approached Carus for pragmatic assistance in their craniological projects without engaging in deeper intellectual exchanges.

Baer and Carus also shared an unethical approach to skull collection. In 1859, Baer informed Retzius of “having stolen several skulls from ossuaries” in Chur, “until a narrow-minded clergyman stood up to me and I finally fled Chur to avoid being imprisoned as a desecrator of the church.”^27^ Carus, for his part, not only relied on his son Albert to secretly loot ancient Roman catacombs for cranial material against the will of the local custodian (Carus 1856, 335) but also enlisted the help of French colonial doctor Jean Louis Geneviève Guyon to obtain the skulls of deceased Muslims, which went against prevailing burial ethics. In a letter to Carus, Guyon acknowledged moral problems in object acquisition: “These kinds of objects are not always easy to obtain, because of the respect that Muslims have for their dead.”^28^ With due patience, however, he managed to acquire a sample of “Arabic” skulls, which he then dispatched to Carus.^29^ The correspondence thus demonstrates that pragmatic interests often outweighed intellectual antipathies.

The engagement of Retzius and Wagner with Carus during the 1840s, along with Lucae’s appraisal of Carus’s skull measurement methods throughout the 1850s, demonstrates that the latter’s anthropological approach was not dismissed from the outset. During this period of consolidation, anthropologists appeared open to a variety of methodological frameworks. The eventual rejection of Carus’s physiognomic craniology and its foundation—the Romantic vertebrae theory—reflects the continuous advance of experimental methods in anatomy and physiology. By the 1860s, Paul Broca and Rudolf Virchow were steering anthropology toward a more rigorous biological science, establishing the “metrical-morphological method” (Martin 2021: 75). This approach reified Retzius’s “distinction between broad and long skulls … as the key index of European race classification” (McMahon 2019: 35). While no less prone to racism, their work was a far cry from Carus’s anthropological writings that blended craniometry with physiognomic symbolism. Notably, nearly all letters by fellow anthropologists spoke of Carus’s esteemed reputation and contributions to the advancement of science. His standing as a leading naturalist of the Romantic period might explain why, despite significant intellectual incompatibilities, he was able to maintain and expand his craniological network throughout the 1860s. While many disagreed with his methods and some of his approaches, his interpretative framework could not be avoided.

## Craniology for Aristocrats

At the age of 49, Carus was a latecomer to craniology and his studies were driven more by a distaste for Gall’s phrenology than by an engagement with contemporary anthropological research. In his memoirs, he attributed his foray into the field to a spontaneous idea: “Finally, the decisive idea dawned on me (quite spontaneously during a soirée where I talked about Gall’s phrenology) concerning the wonderful but hard-to-understand skull, and this idea then stimulated my mind to thousands of possible applications” (Carus 1866a: 107). The birth of Carus’s craniology from an evening gathering, which was likely attended by members of the royal court and the upper Dresden bourgeoisie, helps place Carus’s anthropology in perspective.^30^ Contact with aristocrats and the upper bourgeoisie was crucial to his craniological network. His hagiographic approach encompassed skulls from scientific and literary “geniuses,” as well as many from deceased aristocrats and statesmen,^31^ underscoring the salience of “great men” (Gamper 2016) for Carus’s anthropological thinking. As a result, perhaps, the upper echelons of society seemed more receptive to Carus’s craniological theories than his scientific peers. Carus kept his collection as an arcanum of science, guiding only invited guests through it and keeping it disclosed from the public.

In terms of collecting, Carus leveraged his connections at European courts to inquire about craniological materials. For example, Wenzel Emmanuel Ulrich, a contact at the Vienna court, investigated the whereabouts of Beethoven’s death mask on Carus’s behalf and mentioned an English aristocrat who was about to purchase it. Ulrich offered to introduce Carus as a co-bidder, suggesting that he had a chance at acquiring it.^32^ Similarly, Georg Friedrich von Jäger, the custodian at the Royal Natural Cabinet in Stuttgart, sent a plaster cast of a microcephalic boy’s skull at a time when Carus was particularly interested in so-called Cretin skulls. Exchanges like these usually served mutual interests. In return for the microcephalic skull, Jäger requested that Carus present a geological treatise he had authored to the King of Saxony.^33^ Carus also tapped into the diplomatic circles of the Royal Saxonian court to obtain skulls. In 1856, he asked Friedrich Karl Vitzthum von Eckstädt, the Saxonian envoy to London, to procure two plaster casts of skulls from the British Museum. Initially, Vitzthum assured Carus that replicas of a Babylonian gold mask and an Assyrian skull could be obtained “without the slightest difficulty.”^34^ However, he later retracted his promise upon realizing that the objects were too fragile to allow for casting.^35^ A year later, Vitzthum reported on the display of Julia Pastrana (Garland-Thomson 2012) at a London fair, providing a detailed physical description of her physical anomalies that would undoubtedly pique Carus’s interest.^36^

In 1842, Carus was visited by Prince Herrmann von Pückler-Muskau and Countess Ida von Hahn-Hahn, a German poet. Describing them as “original figures of the modern world,” Carus considered them “living objects of anthropology” and took craniological measurements and plaster casts of their heads (Carus 1866a: 148). That same year, Grand Duchess Elena Pavlovna of Russia expressed a keen interest in Carus’s craniological collection. After observing the objects at his residence, she arranged for plaster casts of the death masks of Pushkin, Peter the Great, and Carl XII of Sweden to be sent to him from St. Petersburg. Carus was deeply impressed by the Grand Duchess, recalling in his memoirs “the image of this uniquely beautiful woman and the grace, liveliness and subtlety of her speech.” He found it insightful to see her and her daughters “amidst my skulls and skeletons! The image of life among images of death!” (Carus 1866a: 170–71). Such analogies are common in Carus’s memoirs, suggesting that his craniological collection inspired perhaps less scientific inquiry than an existential reflection upon life and death.

Carus’s tendency to draw analogies between cranial characteristics and the greater order of things also emerged during a meeting with his longtime friend and patient, Countess Ida von Lüttichau. After attending a performance of *Othello* in 1853 starring the African-American tragedian Ira Aldridge in Dresden, Carus wished to obtain a plaster cast of Aldridge’s head for his collection. He believed that a character like Othello could be portrayed only by an actor with a “receding frontal bone,” a feature that he generally associated with so-called people of the night. Aldridge’s appearance sparked a “long conversation” with von Lüttichau about “differences in character, and more broadly, human diversity” (Carus 1866b: 94). In line with his physiognomic analogies, Carus envisioned two types of human souls: rounded (*gerundete*) and angular (*eckige*), which he believed did not mix well. “The rounded figure can never cling to the angular one without being pressed by it … and the angular one actually always envies the round one because in the end all living things have precisely this form and the primal phenomenon of organic creation will always and forever be the cell” (Carus 1866b: 95) We may safely assume that Carus classified cranial features like Aldridge’s as “angular,” whereas the “rounded” type was most perfectly embodied in exceptional “people of the daylight,” such as Friedrich Schiller, whose presumed skull, Carus noted, displayed “fine roundedness and development of the entire skull” (Carus 1843–1845).^37^

Aristocratic visits to Carus’s skull collection extended to the highest echelons of society. In 1855, the Royal Saxonian family visited Carus’s residence, where he eagerly guided them through the treasures of his collection. The royal family “wandered through my entire house with the most pleasant friendliness, looked attentively at all the art treasures contained here and there, but then spent a particularly long time in my cranioscopic … collection, where I explained all the curiosities to them in the best possible way” (Carus 1866b: 132). Exhibiting artworks alongside the skulls and masks of deceased royals, poets, scientists, statesmen, criminals, mentally-disabled people, Europeans and non-Europeans, Carus’s collection more closely resembled an early modern *Wunderkammer* than the systematic collections found elsewhere. Given Dresden’s long tradition of such displays (Watanabe-O’Kelly 2002: 212–20), Carus’s eclectic array of objects likely found a more receptive audience among social circles traditionally accustomed to such collection practices.

The fact that his skull collection was so often visited by kings and princesses, barons and duchesses aptly reflects his aristocratic attitude, which translated into a general rejection of public access to science. As early as 1835, when he encountered his first cranial specimens in Cuvier’s anatomical gallery, Carus was repelled by public displays of natural history objects. To him, “exposing collections of purely scientific importance to the gawking eyes of a curious mob [*Volksmasse*]” was “to take liberalism too far.” As he exclaimed, with religious pathos, “It does not behoove the priest of nature to publish everything!” Instead, science was best kept as a sacred arcanum, lest “the people lose their awe of the profundity of science” (Carus 1835: 179). He “confessed” that he was “most disgusted to see women from the people, soldiers and boys roaming around in these rooms …, making trivial remarks about the most instructive specimens.” (Carus 1835: 180) When he was finally admitted to the private room containing cranial objects, he was very relieved that he and his company “stayed there a little longer” (Carus 1835: 181). Here too, Carus’s approach to science was at odds with that of his anthropological contemporaries. Karl Ernst von Baer, for example, considered the popularization of science both a goal and a duty, by delivering, among other things, anatomical lectures to “a mixed, nonmedical public” (von Baer 1866, 288) or publishing popular articles in local newspapers and calendars (von Baer 1866: 234, 510).

## Well-Connected, Intellectually Marginalized

This paper aimed to illuminate the complex role of Carl Gustav Carus within the burgeoning field of physical anthropology through an analysis of his craniological network. Despite Carus’s strong ties to the anatomical elites of Europe, which enabled him to amass a large collection of cranial objects, his cranioscopic method failed to have a significant impact on the academic landscape. The limited appeal of his approach can be attributed both to the waning interest in Romantic *Naturphilosphie*—specifically, the vertebrae theory of the skull—and to Carus’s reluctance to engage more deeply with the methodologies of his contemporaries. The period preceding the institutionalization of physical anthropology in the 1860s was somewhat more receptive to *naturphilosophisch* craniology, as evidenced by the efforts of Anders Retzius and Rudolph Wagner and the influence of Carus’s vertebrae theory on the anthropological works of Johann Lucae. Ultimately, however, Carus’s approach to anthropology can be considered untimely. While there is no direct relationship between his craniological theory and his display of cranial objects, both mirrored an aristocratic attitude toward science. Throughout his life, Carus chose not to publicize his collection, limiting access to intellectuals and social elites. Aside from fellow anatomists, naturalists, and zoologists, his connections with various European courts were crucial for acquiring new craniological objects. This aristocratic approach toward science is further reflected in the importance Carus attributed to the skulls and casts of “great men,” which he regarded as equally crucial to his anthropological theory as the skulls of non-European people. This focus helped him reinforce the notion that the European “genius” represented the pinnacle of human civilization.

## Supplementary Information

The supplementary Excel spreadsheet offers insights into the craniological network of Carl Gustav Carus (1789–1869), based primarily on an analysis of his correspondence housed at the Sächsische Staats-, Landes- und Universitätsbibliothek Dresden. It is further enriched by materials from other private collections (e.g., Karl Ernst von Baer, Rudolf Wagner) as well as by Carus’s published works. The dataset outlines collection data for objects listed in the second edition of the Verzeichnis der Cranioskopischen und Chirognomischen Sammlungen des Geheimen Rath Dr. C. G. Carus (Dresden, 1867). Wherever possible, connections between source materials and specific catalogue item numbers have been suggested. However, these links should be regarded as tentative, as the correspondence provides limited evidence and many catalogue entries share identical or similar names. In addition, the dataset includes references to items that were not actually exchanged but only mentioned in the context of offers or requests—either from Carus or addressed to him. The primary focus is on mapping the collector’s network, which is why a second table lists all identified correspondents.

## Footnotes

1 The meeting included nine self-designated anthropologists including Ernst von Baer, Rudolf Wagner, and Carl Georg Bergmann (anatomist and physiologist); Johann Christian Lucae, Jacob Henle, and Ernst Heinrich Weber (anatomists); Willem Vrolik (anatomist and zoologist); Wilhelm Krause (pathologist); and Georg Meisner (physiologist). Other scholars invited but unable to attend included Alexander Ecker and Hermann Schaafhausen (anatomists); Jan van der Hoeven and Pieter Harting (zoologists); Karl Scherzer (explorer); and the naval physician Eduard Schwarz (Querner 1986: 295).

2 Universitätsbibliothek Gießen, Nachl. Baer, Briefe 25, fol. 295–96: Karl Ernst von Baer to Anders Adolph Retzius, February 7, 1858, fol. 296r. In the German original, Baer speaks of “Jucker-Pferden,” which were half-bred Hungarian horses used for light carriages (Meyers 1905–1909, 326). All translations of German quotations into English by Stephan Strunz.

3 Ibid.

4 Universitätsbibliothek Gießen, Nachl. Baer, Briefe 20, fol. 281–82: Rudolf Wagner to Karl Ernst von Baer, March 3, 1861, fol. 282r.

5 Ibid., fol. 285r.

6 On provenance research at the University of Leipzig see the project “Rechercen zu Provenienz und Sammler:innen der afrikanischen sowie süd- und mittelamerikanischen human remains der Leipziger Schädelsammlung“, https://www.proveana.de/de/link/pro00000261, last accessed June 27, 2025. As of September 2024, the Dresden Museum of Ethnology has not provided written clarification on ongoing provenance research and potential restitution of the Carus collection, despite repeated attempts to make contact. Communication with the custodian in charge has been difficult, so that the paper does not include information concerning the current state of the collection.

7 The unpublished letters to Carus can be found here: Sächsische Landesbibliothek—Staats- und Universitätsbibliothek (in the following: SLUB), Mscr.Dresd.h.45.

8 Niedersächsische Staats- und Universitätsbibliothek Göttingen, Cod. Ms. R. Wagner II: C. G. Carus; Universitätsbibliothek Gießen, Nachl. Baer.

9 SLUB, Mscr.Dresd.h.45. Bd. 6, Nr. 78: Karl Vogel to Carl Gustav Carus, September 4, 1863.

10 SLUB, Mscr.Dresd.h.45. Bd. 2, Nr. 48: Daniel Friedrich Eschricht to Carl Gustav Carus, December 8, 1859.

11 SLUB, Mscr.Dresd.h.45. Bd. 2, Nr. 46: Daniel Friedrich Eschricht to Carl Gustav Carus, September 9, 1840.

12 Ibid.

13 SLUB, Mscr.Dresd.h.45. Bd. 2, Nr. 47: Daniel Frederik Eschricht to Carl Gustav Carus, February 2, 1841.

14 SLUB, Mscr.Dresd.h.45. Bd. 5, Nr. 137: Anders Retzius to Carl Gustav Carus, May 5, 1842.

15 SLUB, Mscr.Dresd.h.45. Bd. 5, Nr. 138: Anders Retzius to Carl Gustav Carus, July 3, 1845.

16 Ibid.

17 Universitätbibliothek Gießen, Nachl. Baer, Briefe 17: Anders Retzius to Karl Ernst v. Baer, March 7, 1859, fol. 672r.

18 SLUB, Mscr.Dresd.h.45. Bd. 6, Nr. 82: Rudolph Wagner to Carl Gustav Carus, May 29, 1843.

19 Ibid.

20 Niedersächsische Staats- und Universitätsbibliothek Göttingen, Cod. Ms. R. Wagner II: C. G. Carus: Carl Gustav Carus to Rudolph Wagner, June 13, 1843.

21 SLUB, Mscr.Dresd.h.45. Bd. 6, Nr. 82: Rudolph Wagner to Carl Gustav Carus, May 29, 1843.

22 SLUB, Mscr.Dresd.h.45. Bd. 4, Nr. 34: Johann Christian Gustav Lucae to Carl Gustav Carus, November 11, 1846.

23 Ibid.

24 SLUB, Mscr.Dresd.h.45. Bd. 3, Nr. 89: Alfred Higgins to Carl Gustav Carus, August 19, 1863.

25 SLUB, Mscr.Dresd.h.45. Bd. 1, Nr. 24: Karl Ernst v. Baer to Carl Gustav Carus, July 28, 1858.

26 Ibid.

27 Universitätsbibliothek Gießen, Nachl. Baer, Briefe 25: Karl Ernst v. Baer to Anders Retzius, February 2–19, 1859, fol. 295r.

28 SLUB, Mscr.Dresd.h.45,Bd. 2, Nr. 141: Jean Louis Geneviève Guyon to Carl Gustav Carus, March 1st, 1848.

29 Ibid.

30 The close relationship of Carus to the Royal House of Wettin and its evening gatherings has been stressed by Albert of Saxony, a descendant of the House of Wettin (2005).

31 To cite just a few examples from Carus’s catalogue, the collection contained plaster casts of the skulls of Frederick the Great, Charles Maurice de Talleyrand-Périgord, Robespierre, Carl XII. of Sweden, Lorenzo di Medici, Peter the Great, Benjamin Franklin, Karl August von Hardenberg, Queen Louise of Prussia, Antoni Radziwiłł, and Anna Amalia of Weimar (Carus 1867 [1863]).

32 SLUB, Mscr.Dresd.h.45. Bd. 6, Nr. 62: Wenzel Emmanuel Ulrich to Carl Gustav Carus, April 1, 1846.

33 SLUB, Mscr. Dresd.h.45. Bd. 3, Nr. 103: Georg Friedrich von Jäger to Carl Gustav Carus, November 16, 1848.

34 SLUB, Mscr.Dresd.h.45. Bd. 6, Nr. 71: Karl Friedrich Vitzthum von Eckstädt to Carl Gustav Carus, February 24, 1846.

35 SLUB, Mscr.Dresd.h.45. Bd. 6, Nr. 72: Karl Friedrich Vitzthum von Eckstädt to Carl Gustav Carus, March 21, 1856.

36 SLUB, Mscr.Dresd.h.45. Bd. 6, Nr. 73: Karl Friedrich Vitzthum von Eckstädt to Carl Gustav Carus, July 10, 1857.

37 Ironically, the myth of Schiller’s supposedly perfectly rounded skull was later debunked by Hermann Welcker, who found that it must have belonged to another person (Welcker 1883: 40).
